# The Glutamate System as a Crucial Regulator of CNS Toxicity and Survival of HIV Reservoirs

**DOI:** 10.3389/fcimb.2020.00261

**Published:** 2020-06-24

**Authors:** Anna Maria Gorska, Eliseo A. Eugenin

**Affiliations:** Department of Neuroscience, Cell Biology, and Anatomy, The University of Texas Medical Branch, Galveston, TX, United States

**Keywords:** reservoirs, cure, NeuroHIV, dementia, glutamate, glutamine, HIV

## Abstract

Glutamate (Glu) is the most abundant excitatory neurotransmitter in the central nervous system (CNS). HIV-1 and viral proteins compromise glutamate synaptic transmission, resulting in poor cell-to-cell signaling and bystander toxicity. In this study, we identified that myeloid HIV-1-brain reservoirs survive in Glu and glutamine (Gln) as a major source of energy. Thus, we found a link between synaptic compromise, metabolomics of viral reservoirs, and viral persistence. In the current manuscript we will discuss all these interactions and the potential to achieve eradication and cure using this unique metabolic profile.

## Introduction

Neurons are not able to perform *de novo* synthesis of the neurotransmitters glutamate (Glu) and gamma-aminobutyric acid (GABA) from glucose due to the lack of enzymes involved in their metabolism. Thus, a multicellular metabolic pathway known as the Glu/GABA-glutamine (Gln) cycle maintains the balance among these metabolites to support neuronal synaptic transmission (Bak et al., 2006).

Glu is the most abundant excitatory neurotransmitter in the central nervous system (CNS) (Zhou and Danbolt, 2014), and GABA is considered an inhibitory neurotransmitter in adulthood (McCormick, 1989; Harris-Warrick, 2005). A balance between these two neurotransmitters plays a critical role in several brain functions including learning and memory, development, pain, synaptogenesis, motor stimuli, and synaptic signaling (Petroff, 2002; Allen et al., 2004; Bak et al., 2006; Bonansco and Fuenzalida, 2016; Ford et al., 2017). Alterations in this balance have been associated with brain damage and several neurodegenerative diseases including Alzheimer's, Parkinson's, and NeuroHIV. In the particular case of NeuroHIV, the equilibrium of Glu/GABA/Gln is altered and contributes to neuronal and glial dysfunction as well as to cognitive impairment observed in at least half of the HIV-1-infected population, even in the current antiretroviral treatment (ART) era (Sailasuta et al., 2009; Cohen et al., 2010; Ernst et al., 2010; Young et al., 2014; Mohamed et al., 2018; Cysique et al., 2019). Briefly, the pathogenesis of NeuroHIV involves the early (7–10 days post-injection) transmigration of leukocytes carrying the virus across the blood-brain barrier (BBB) using chemokine gradients only sensed by HIV-1-infected monocytes due to their enhanced expression of key chemokine receptors such as CCR2 (Eugenin et al., 2006; Williams et al., 2013). Upon crossing the BBB, the few transmigrated HIV-1-infected leukocytes infect CNS resident cells such as microglia, macrophages, and a small population of astrocytes. If systemic HIV-1 replication is not controlled by ART, localized HIV-1-CNS replication and infection results in HIV-1 encephalitis and dementia (Gatanaga et al., 1999; Bingham et al., 2011; Gelman et al., 2013; Gelman, 2015; de Almeida et al., 2017; Mangus et al., 2018). However, in the current ART era, CNS damage is mild due to controlled peripheral and CNS replication as well as limited HIV-1 infection; despite this, 50% of the HIV-1-infected individuals still show significant signs of cognitive impairment, but the mechanism of CNS dysfunction is unknown (Eggers et al., 2017; Yoshimura, 2017; Bandera et al., 2019; Fernandes and Pulliam, 2019; Kim-Chang et al., 2019; Paul, 2019; Portilla et al., 2019; Swinton et al., 2019; Angelovich et al., 2020). Several groups have proposed that CNS damage in the current ART era corresponds to a combination of HIV-1 reservoirs within the brain, low level expression and secretion of viral proteins, as well as associated inflammation (Wong and Yukl, 2016; Veenstra et al., 2017, 2019). However, the nature and size of the viral reservoir within the CNS under effective ART is unknown (Churchill et al., 2006, 2009; Eugenin et al., 2011a; Russell et al., 2017; Al-Harti et al., 2018; Ko et al., 2019; Wallet et al., 2019). Our laboratory demonstrated that myeloid and glial cells within the CNS are viral reservoirs. These few viral reservoirs, despite the low to undetectable viral replication, are able to amplify toxicity and inflammation to neighboring uninfected cells by a gap junction, hemichannel, and tunneling nanotube mediated mechanism (Eugenin et al., 2009a,b; Berman et al., 2016; Malik and Eugenin, 2016, 2019; Ariazi et al., 2017; Okafo et al., 2017; Valdebenito et al., 2018).

Another potential mechanism of toxicity is mediated by the low-level production of viral proteins, not blocked by ART, and subsequent secretion into neighboring cells such as neurons and glia (Nath, 2002; Kovalevich and Langford, 2012; Sami Saribas et al., 2017). However, the extent and concentrations of viral proteins in the CNS and other tissues are unknown, but nanograms/ml of viral proteins (Nef, Tat) were detected in the serum or plasma of HIV-1-positive individuals (Westendorp et al., 1995; Goldstein, 1996; Xiao et al., 2000). Almost all HIV-1 proteins are neuro- or glial-toxic. For example, neurotoxicity has been described for several viral proteins including gp120 and the transactivator of transcription (Tat) (Table 1), as well as for host factors such as TNF-α, IL-1β, and IL-6 released from latently HIV-1-infected or -activated cells (Koller et al., 2001; Zhou et al., 2017). In addition, several ART drugs have been demonstrated to generate toxicity on their own (Brier et al., 2015; Underwood et al., 2015; Latronico et al., 2018). Thus, the combination of HIV-1-infection, low-level secretion of viral proteins, and ART toxicity probably contribute to CNS dysfunction.

**Table 1:** Effect of HIV-1 proteins on neurotoxicity induced by glutamatergic system dysregulation.

**HIV-1 protein**	**Effect on Glu-related neurotoxicity**	**References**
Gp120	Induce synaptodendritic degeneration by activation of co-receptors CXCR4 and CXCR5.	Kaul et al., 2007
Gp120	Induces a massive calcium release and changes in the morphology of neuronal mitochondria.	Avdoshina et al., 2016; Rozzi et al., 2017
Gp120	Increase the synaptic damage *via* NMDAR based on enhanced of NR2A- and NR2B-mediate EPSCs.	Yang et al., 2013; Zhou et al., 2017
Gp120	In HIV/gp120-tg mice pharmacological blockade of NMDAR was protective against gp120 neurotoxic properties involved in causing neuronal damage and synaptic loss.	Nakanishi et al., 2016
Gp120	Modulate the intracellular trafficking and surface clustering of NMDAR.	Scott et al., 2003; Xu et al., 2011; Ru and Tang, 2016
Gp120	Potentiate neuronal cell death and prolonged increased in level of intracellular calcium induced by Tat.	Nath et al., 2000
Gp120	In primary human astrocytes HIV-1 and gp120 impaired the clearance of Glu by reducing the expression of EAAT2.	Wang et al., 2003; Melendez et al., 2016
Gp120	Reduced astroglial cell viability. In addition, gp120 reduced both Gln concentration in astroglial cell supernatants and GS expression.	Visalli et al., 2007
Gp120	Increase of AEG-1 expression in gp120-treated astrocytes, followed by impaired Glu homeostasis due to down-regulation of EAAT2 in astrocytes.	Kang et al., 2005; Lee et al., 2011
Tat	Stimulate NMDARs by direct cysteine-cysteine interaction of Tat with the extracellular domain of the receptor.	Prendergast et al., 2002; Li et al., 2008
Tat	Promotes the phosphorylation of the NMDAR and triggers the calcium efflux and receptors stimulation.	Haughey et al., 2001
Tat	Induce cell death in human neuroblastoma cells (SH-SY5Y) by *a* mechanism involving H_2_O_2_ release and NMDAR activity.	Capone et al., 2013
Tat	Amino acids 31–61 of Tat are necessary to cause neurotoxicity. Tat Δ31–61 mutant protein were not able to bind to the NMDAR and induce neurotoxicity.	Li et al., 2008
Tat	The changes in Tat protein structure prevent the interaction with the NMDAR, also the immune complex of the mutant Tat or nitrosylated Tat and anti-Tat antibody block neurotoxicity caused by NMDAR agonist.	Rumbaugh et al., 2013
Tat	Tat binding to LRP on the neuron's membrane initiate the formation of a macro molecular complex among tat-LRP-PSD-95 (as an intracellular adaptor protein)-NMDAR. LRP antagonist, blocked the Tat-dependent NMDAR potentiations.	Eugenin et al., 2007; Krogh et al., 2014b
Tat	Application of Tat into human fetal neurons result in calcium release *via* stimulation of IP_3_ receptor and activation of neuronal nitric oxide synthetase (nNOS).	Haughey et al., 1999
Tat	Induced the macrophage/microglia activation and microglia-mediate neurotoxicity, caused by Tat-dependent activation of NADPH oxidase.	Minghetti et al., 2004; Turchan-Cholewo et al., 2009
Tat	Induced the dose-dependent Glu release from microglial which was associated with increased expression of the X_c−_ glutamate-cystine antiporter, and this effect was blocked by NADPH oxidase and glutamate-cystine inhibitors.	Barger et al., 2007; Gupta et al., 2010
Tat	Induced the Nrf2 activation and X_c−_ system up-regulation, which could be a potential source of excitotoxicity induced my Glu release.	Fogal et al., 2007; Bridges et al., 2012a,b; Mastrantonio et al., 2019
Vpr	Induced apoptosis in human neuronal cells *via* activation of caspase-8.	Patel et al., 2000
Vpr	Decreased antioxidants pool in astrocytes *via* increased production of reactive oxygen species due to an increase in the level of oxidized glutathione (GSSG).	Ferrucci et al., 2012
Vpr	Up-regulates the GLS isoform C expression, resulting in an increased level of Glu in a media of HIV-1-infected macrophages.	Erdmann et al., 2009; Datta et al., 2016
Vpr	Altering protein involved in glycolytic and citrate pathways in human derivative macrophages.	Barrero et al., 2013
Vpr	Glu production and release is mediated by glucose-dependent metabolism following by activation of glycolytic and TCA cycle in Vpr overexpressing macrophages.	Datta et al., 2016

Another accepted mechanism of toxicity is Glu-mediated toxicity. Glu is a neurotransmitter dysregulated in HIV-1 infection that can contribute to HIV-associated neurocognitive disorder (HAND) (Huang et al., 2011; Potter et al., 2013; Vazquez-Santiago et al., 2014). In addition to synaptic dysregulation, a supplemental source of Glu in HIV-1 conditions is the persistently activated resident microglia and invading macrophages, which have been shown to increase Glu synthesis and to release into the extracellular space (Potter et al., 2013; Wu et al., 2015). Furthermore, in several cell types, HIV-1 infection leads to mitochondrial membrane destabilization and the release of phosphate-activated glutaminase (PAG) from the mitochondria matrix into the cytosol through the transition pore (Erdmann et al., 2007, 2009; Huang et al., 2011; Tian et al., 2012). Glutaminase (GLS) is an enzyme responsible for *de novo* synthesis of Glu from Gln resulting in the increased levels of intracellular and extracellular Glu due to mitochondrial dysfunction.

In individuals with NeuroHIV the extracellular level of Glu is elevated in cerebrospinal fluid (CSF) and plasma in correlation with severity of brain atrophy and dementia (Ferrarese et al., 2001; Cassol et al., 2014; Anderson et al., 2015). However, MRI/MRS imaging data are controversial. Some reports indicated that HIV-1-infected individuals with dementia have decreased levels of Glu in frontal gray and white matter as well as the basal ganglia in correlation with cognitive deficits (Sailasuta et al., 2009; Ernst et al., 2010; Mohamed et al., 2018). In contrast, other publications using MRS indicate that Glu is increased in the frontal white and gray matter and basal ganglia in HIV-1-infected individuals before and after initiation of ART as compared to uninfected individuals. However, treatment of HIV-1-infected individuals for extended periods of time with ART results in the reduction of extracellular levels of Glu, suggesting a positive effect of ART treatment (Young et al., 2014). Thus, most of these changes in Glu assessed by brain imaging are probably due to individual-to-individual variability, HIV-1 time course, HIV-1 replication, time of infection, ART, and several other potential CNS complications. Despite the mechanism of Glu dysregulation, we recently identified that glial and myeloid viral reservoirs within the brain generate energy from unusual sources of energy such as Glu and Gln, both of which are highly abundant in the brain, providing them with an almost endless source of energy. We recently demonstrated that blocking some of these metabolic pathways results in the effective killing of viral reservoirs, even in the absence of HIV-1 reactivation.

In this review, we will describe the overall Glu/GABA synaptic system, its dysregulation, and the role of HIV-1 in CNS dysfunction with a major focus on viral reservoirs.

## An Introduction to the Glutamate/Gamma-Aminobutyric Acid Neurotransmitter System

Glu binding activates ionotropic and metabotropic receptors expressed on neuronal, glial, and immune cells (Zhou and Danbolt, 2014). Membrane-specific Glu receptors (GluRs) are expressed in neurons and glial cells, and they mediate most, but not all, excitatory and inhibitory effects (D'Antoni et al., 2008; Petralia, 2012; Skowronska et al., 2019). The activation of ionotropic Glu receptors mediates fast excitatory synaptic transmission, whereas the metabotropic receptors play a modulatory role (Lau and Tymianski, 2010).

Ionotropic Glu receptors (iGluRs) are proteins composed of different subunits that form ion channels. They are divided into three groups based on their structural similarities and named according to the type of agonist that activates them: amino-3- hydroxy-5-methylisoxazole-4-propionate (AMPAR), N-methyl- D-aspartate (NMDAR), and 2-carboxy-3-carboxymethyl-4-isopropenylpyrrolidine (kainate, KA) receptors (KAR) (Traynelis et al., 2010). The NMDAR family is composed of seven receptor families. They are divided in three main subunits, GluN1, GluN2, and GluN3, which present different subtypes: GluN2–N2A, N2B, N2C, N2D; GluN3–N3A, N3B (Kumar, 2015). For a functional channel, an NR1 and an NR2 subunit are required. Glu binds to the NR2 subunit while the co-agonist, glycine, binds to the NR1 subunit. The binding of the co-agonist enables the removal of the magnesium blockade (Guo et al., 2017; Gibb et al., 2018). Similar to NMDAR, the family of AMPA receptors is composed of four subunits, GluR1/2/3/4, and the GluR2 subunit plays a critical role in Ca^2+^ permeability (Lomeli et al., 1994). The KAR family is composed of two types of subunits: GluR5/6/7 and KA1 and KA2, which form homo- or heterotetramers. Depending on the receptor's subunit composition, the channel will have different electrophysiological properties (Contractor et al., 2011).

Metabotropic Glu receptors (mGluRs) have been subdivided in regard to sequence similarity and signaling into three groups (group I: mGluR 1 and 5; group II: mGluR 2 and 3; and group III: mGluR 4, 6, 7, and 8). The mGluRs are G protein-coupled receptors that are linked to various intracellular second messenger cascades. Group I mGluRs are coupled to G_q_, and signaling through these receptors is carried out by the phospholipase C and stimulation of intracellular calcium (Ca^2+^) release. Receptors belonging to this group (mGluR1 and mGluR5) are located predominantly postsynaptically. However, the presynaptic receptors control Glu release. Thus, depending on the receptor location, they have different activities. The second (mGluR2 and mGluR3) and the third (mGluR4, 6, 7, and 8) group are coupled to G_i/o_ proteins, and they inhibit the activity of adenylate cyclase, resulting in a reduction of the intracellular level of cyclic AMP and the inhibition of protein kinase A (Pilc et al., 2008; Wieronska and Pilc, 2009; Niswender and Conn, 2010). In contrast to group I, receptors from the second and third groups are located predominantly presynaptically, and they inhibit the function of glutamatergic neurons, but the receptors from the third group also activate GABA release (Conn and Pin, 1997).

mGluRs receptors modulate neuronal excitability via regulation of voltage-sensitive calcium channels, G protein-coupled inwardly rectifying potassium (GIRK) channels, and GABA, AMPA, and NMDA receptors (Conn and Pin, 1997). Moreover, in several brain regions, iGluRs were identified on synaptic terminals (i.e., presynaptic receptors) as functional autoreceptors to modulate Glu and GABA release (Duguid and Smart, 2004; Fiszman et al., 2005). Interestingly, all these receptors are also expressed in immune cells, non-excitable, providing a unique neuroimmune cross talk that still is under examination (Eck et al., 1989; Pacheco et al., 2007; Moroni et al., 2012; Yang et al., 2013) and can further contribute to NeuroHIV development.

### Characteristic of Glutamate Transporters

The release of intracellular Glu into the extracellular space can be achieved by four different mechanisms: (1) calcium-dependent exocytosis of neurotransmitter from vesicular storage; (2) reverse action of Glu transporters located pre- and postsynaptically on neurons and astrocytes plasma membrane; (3) reverse cystine/glutamate transporter activity on the plasma membrane; and (4) hemichannels (Allen et al., 2004; Santello et al., 2012; Li et al., 2014).

**Figure 1:**
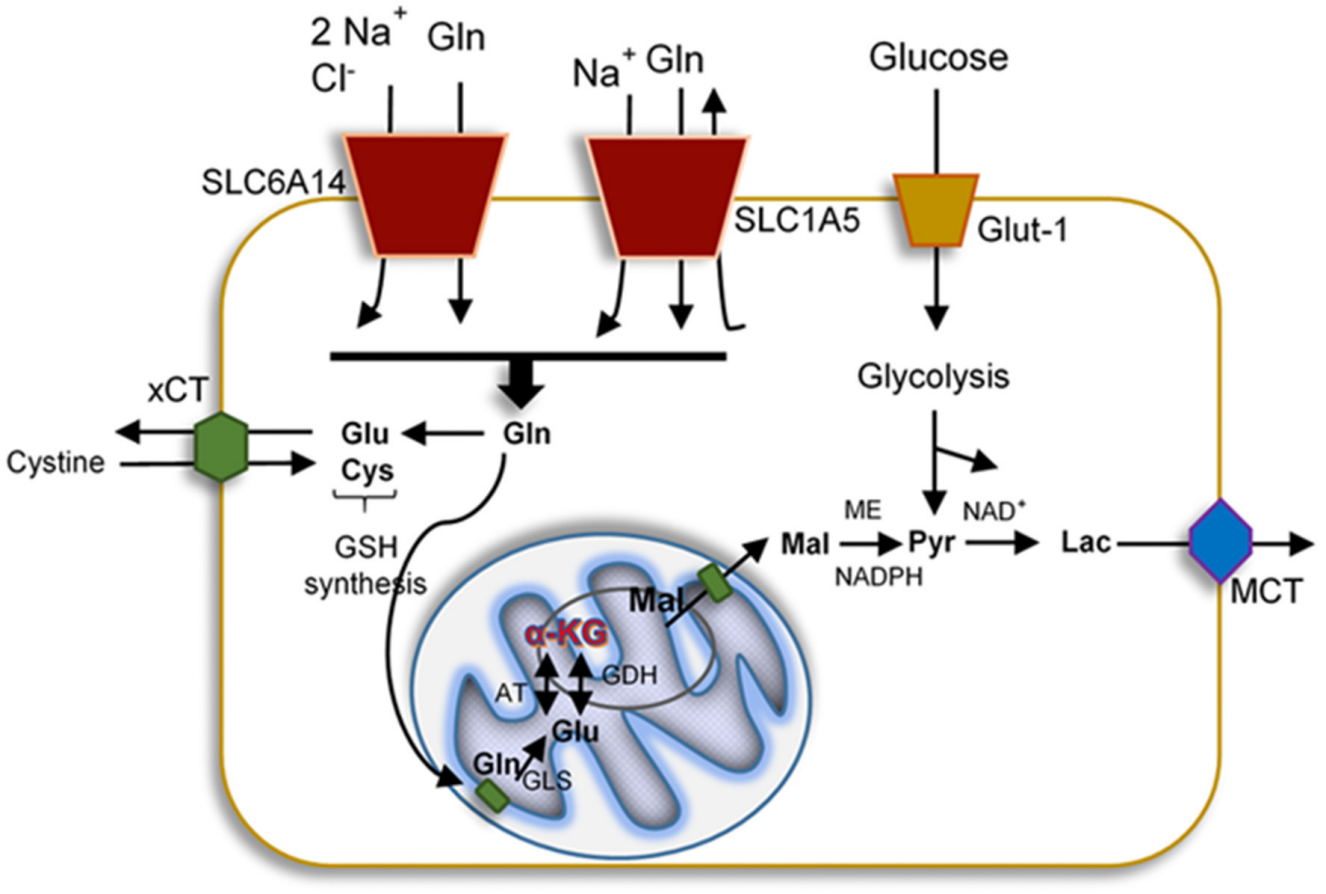
Representation of the uptake and mitochondrial delivery system of glucose, glutamate, and glutamine. Glucose enters the cells by glucose transporter-1 (Glut-1), cystine, Gln, and Glu also are uptake by specific transporters such as cystine/glutamate antiporter (xCT), Na^+^ and Cl^−^-dependent neurotransmitter transporter (SLC6A14), and neutral amino acid transporter (SLC1A5), respectively. All proteins affected by HIV-1 infection. Upon uptake, these molecules can become part of the glycolysis, GSH synthesis, or the TCA. In the brain, metabolism of these mediators generates lactate that exits the cells via the monocarboxylete transporter (MCT).

The first system corresponds to the synaptic regulation of the release and uptake of Glu. Glu in the CNS is synthetized and stored (millimolar concentration) in synaptic vesicles of glutamatergic neurons. The millimolar concentration of Glu in the synaptic cleft is kept in nanomolar range after synaptic stimulation by reverse excitatory amino acid transporters (EAATs). The second system corresponds to Glu transporters belonging to solute carrier family (SLC): Na^+^- dependent (X_AG_) excitatory amino acid transporter (EAAT-1), also known as Na^+^-dependent glutamate/aspartate transporter (GLAST) and EAAT-2 or glutamate transporter 1 (GLT-1). These transporters work based on the maintainence of the Na^+^ gradient between intracellular space and extracellular fluid by Na^+/^K^+^-ATPase. In the CNS, the Glu transporters are located on astrocytes (Rothstein et al., 1996), neurons (Kanai and Hediger, 2004), and the BBB (Kanai and Hediger, 2004), and their main function is to remove the excess of Glu from the synaptic cleft after signal transduction (Nedergaard et al., 2002; Grewer et al., 2008). The third system, X_c−_, is an exchanger of intracellular Glu for extracellular cystine. Cystine is reduced to glutathione (GSH) in the cytoplasm (Lu, 2013). It was published that disruption in *the* X_c−_ the system may lead to an increase of extracellular Glu concentration, which is a source of oxidative stress and cell death (Bridges et al., 2012b; Lewerenz et al., 2013). The fourth system correspond to hemichannels composed of connexin or pannexin proteins. They are expressed on the surface of glial cells including astrocytes and microglia/macrophages, which allow for the rapid intercellular exchange of ions and metabolites. Opening of hemichannels occurs only under stress conditions to serve as a backup system for Glu buffering, calcium wave propagation, and synaptic plasticity (Figure 1). In astrocytes, unopposed hemichannels on astrocytes membrane are responsible for the release of gliotransmitters, including ATP, Glu, nicotinamide adenine dinucleotide (NAD), and D-serine to the extracellular space. Under physiological conditions, hemichannels are in a closed state. However, upon inflammation and damage, hemichannels become open and have been associated with several neurodegenerative disorders (Xing et al., 2019) including NeuroHIV (Eugenin et al., 2011a; Orellana et al., 2014; Berman et al., 2016; Malik and Eugenin, 2016, 2019). Only recently, we identified that circulating levels of ATP can be used to provide an early detection of cognitive impairment in HIV-infected individuals (Velasquez et al., 2020).

### Pathways of Glutamate/Gamma-Aminobutyric Acid Synthesis—Neuro-Glia Interaction

**Figure 2:**
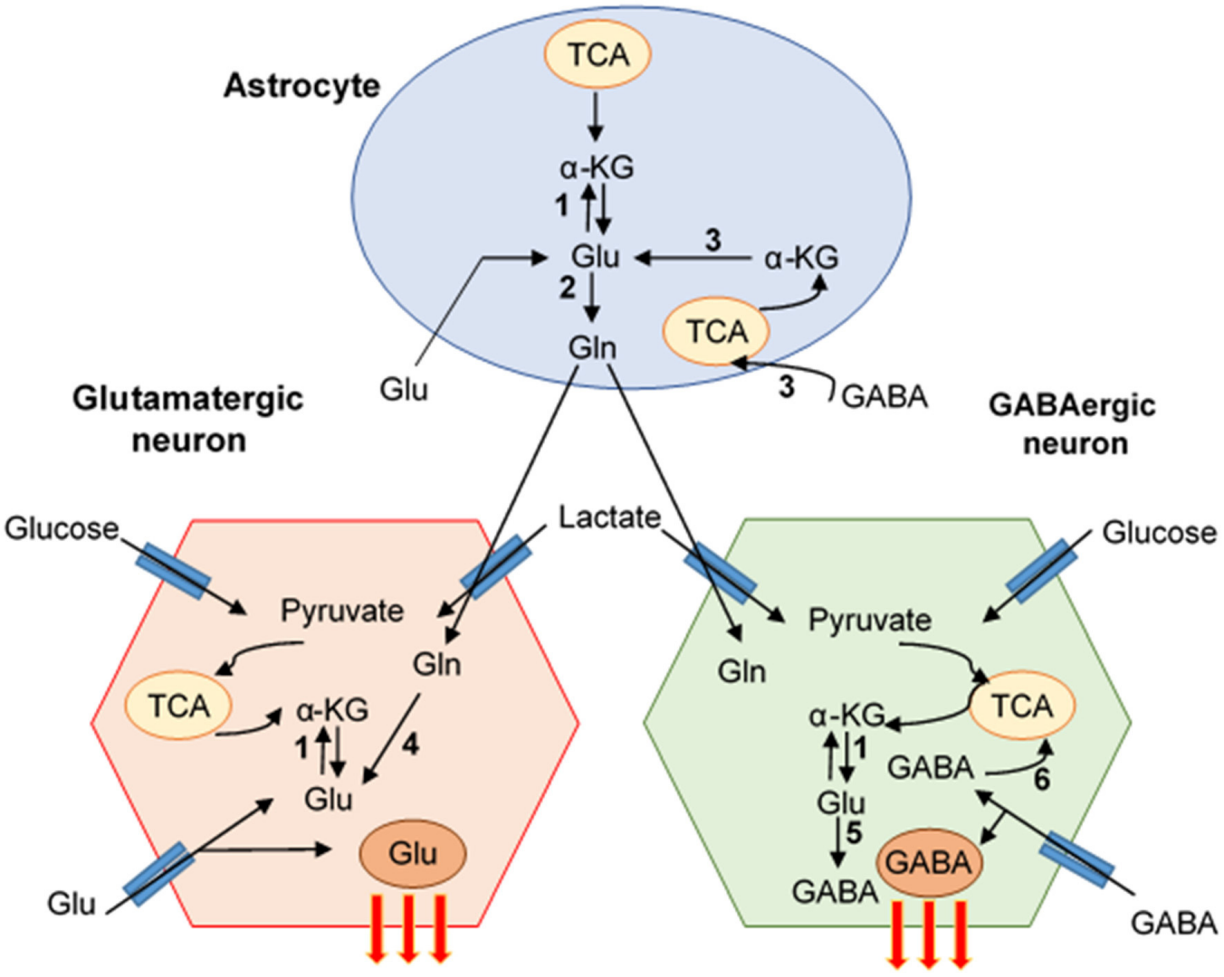
Pathways of Glutamate/GABA synthesis-neuro-glia interaction. The cartoon represents the mutual interaction of astrocyte and glutamate (Glu) and GABA neuron. Glu-GABA cycle enzymes: (1) aminotransferases (AT), (2) glutamine synthetase (GS), (3) glutamate dehydrogenase (GDH)/aspartate aminotransferase (AAT), (4) glutaminase (PAG), (5) glutamate decarboxylase (GAD), (6) GABA transaminase (GABA-T)/succinate-semialdehyde dehydrogenase (SSADH) (Schousboe et al., 2013).

**Figure 3:**
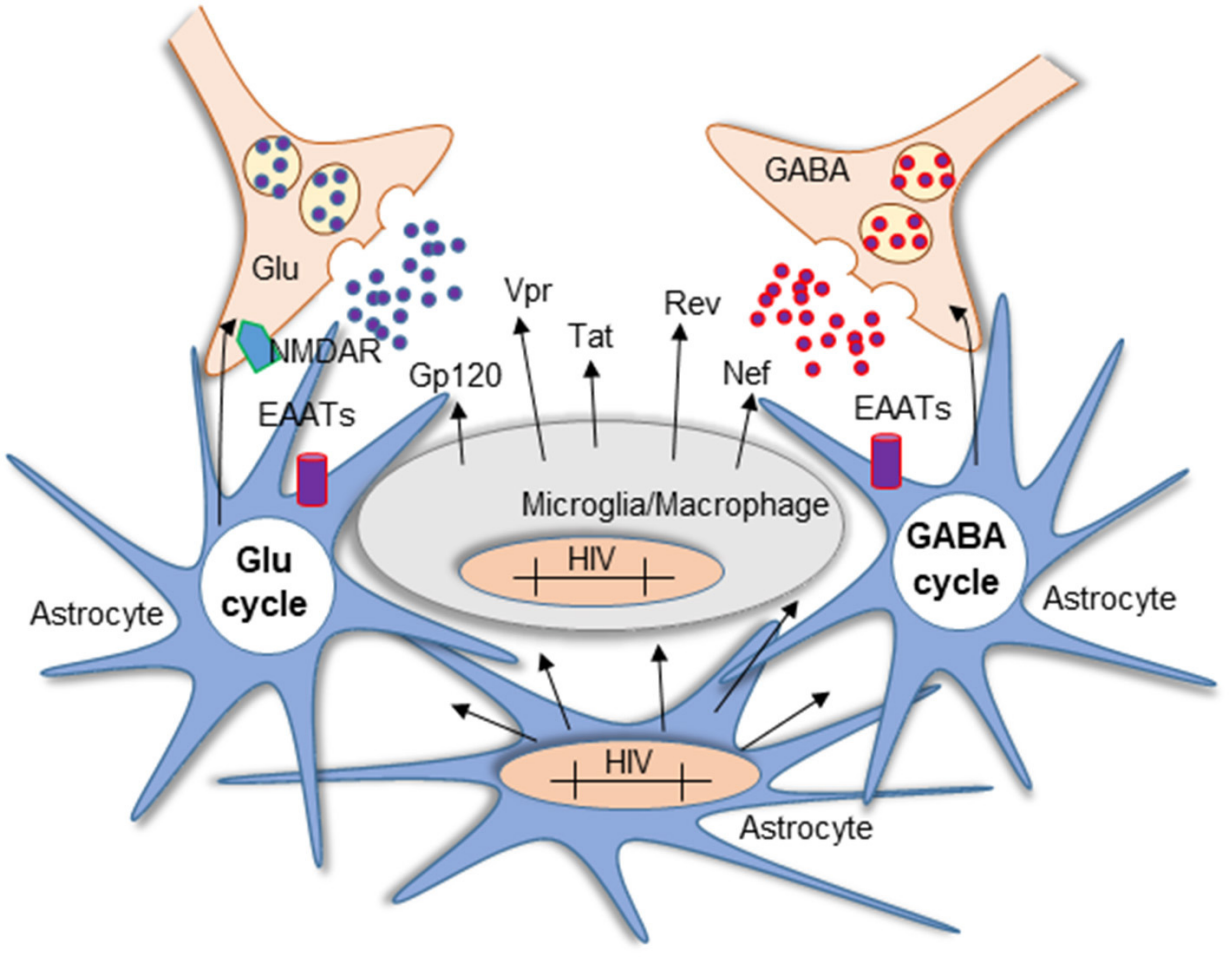
Mechanism of Glu neurotoxicity in NeuroHIV pathogenesis. Glu is a critical neurotransmitter dysregulated in HIV-1 infection and HAND. Imbalance in the Glu/GABA cycle impaired the excitatory/inhibitory state of the neurons resulting in persistent stimulation of NMDARs. The diagram represents the activated state of astrocytes and microglia/macrophages. In the CNS, microglia, macrophages, and a small population of astrocytes become infected and carry HIV-integrated DNA. Although astrocytes cannot produce the virus, they contribute to neurotoxicity mediated by the production of viral proteins that are not blocked by ART and the subsequent secretion into neighboring cells such as neurons and glia. Another source of toxicity is the persistent activation of resident glial cells that increase Glu synthesis and release into the extracellular space.

Neurons cannot synthesize Glu *de novo* due to the lack of expression of glutamine synthetase (GS), which converts Glu to Gln, and pyruvate carboxylase (PC), which converts Gln into Glu (Pardo et al., 2011). In contrast, astrocytes express both enzymes (Bak et al., 2006). After the release of neurotransmitters to the synaptic cleft, the excess of Glu or GABA is removed by neighboring astrocytes using specific transporters and both neurotransmitters are metabolized into Gln via GS or transformed into Glu and then to α-KG to become part of the tricarboxylic acid cycle (TCA cycle, Krebs cycle) (Bak et al., 2006). In glutamatergic neurons, Glu is synthetized on three pathways: (1) from α-KG, a product from the TCA cycle, which is converted by the action of glutamate dehydrogenase (GDH) and aspartate aminotransferase (AAT) into Glu. GDH is a reversible enzyme that can transform a-KG, ammonia, and NADH or NADPH into Glu (Hara et al., 2003). (2) Gln from astrocytes is delivered into neurons by amino acids transporters (SLC6A14 and SLC1A5) and metabolized directly via enzyme glutaminase (GLS)/phosphate-activated GLS (PAG) into Glu. GLS is a main Gln-utilizing enzyme in the brain, converting the Gln to Glu, and it is located on the inner membrane of mitochondria (Holcomb et al., 2000). The two types of GLS are well-characterized as the “kidney type” (GLS1) and “liver type” (GLS2). In the human brain, GLS1 has two isoforms: kidney-type glutaminase (KGA) and glutaminase C, which are expressed in the various cell types including neurons, microglia, macrophages, and astrocytes (Cardona et al., 2015). GLS is known to promote cancer cell proliferation and differentiation (Erickson and Cerione, 2010). Moreover, GLS is an initial enzyme in the glutaminolysis pathway, providing the energy source for protein and lipid synthesis. During glutaminolysis, Gln is converted to Glu and subsequently into α-KG, which then enters the TCA cycle (Figure 1). Also, Glu can be converted into GSH that can protect the cells from oxidative stress and promote their survival. The third pathway (3) of Glu synthesis is from Glu reuptake from extracellular space and transformation into Gln to go back to neurons (Peng et al., 1993; Schousboe et al., 2013) (Figure 2). GABA, similarly to Glu, is metabolized on two pathways: (1) reuptake from extracellular space and incorporation into the TCA cycle and then conversion to α-KG via GABA-transaminase (GABA-T) and succinic semialdehyde dehydrogenase (SSADH), and (2) from Gln, which is converted to Glu, and next via decarboxylation (glutamate decarboxylase enzyme, GAD) to GABA (Schousboe et al., 2013) (Figure 2). The synaptic pool of Glu can be divided into two compartments: (1) intracellular/cytosolic, in which Glu is synthetized from Gln inside glutamatergic neurons via enzyme GLS; and (2) mitochondrial, in which Glu synthesis from Gln is followed by Glu transformation to α-KG, which enters into the TCA cycle. Inside the mitochondria, Gln, even if it is considered as a source of energy, is called the “Trojan horse” because it is not the preferred source of energy. During the metabolism of Glu from Gln, Gln-derived ammonia (${\text{NH}}_{4^{+}}$) is also generated and affects mitochondrial function by increasing the production of ROS and inducing mitochondrial permeability transition (MPT). This process introduces the collapse of the inner mitochondrial membrane and leads to mitochondrial dysfunction and free radicals production (Zoratti et al., 2005; Albrecht and Norenberg, 2006) (Figure 3). Later we will discuss why HIV-1-reservoirs prefer this metabolic pathway to survive for extended periods of time within the CNS. Also, glucose, the main brain energy substrate, is involved in the Glu/GABA synthesis cycle. In astrocytes, glucose is metabolized to lactate and pyruvate, which is a substrate for the TCA cycle, which produces α-KG and Gln, the fundamental substrate for Glu/GABA synthesis (Hertz and Chen, 2017). These tight connections between neurotransmission, neurotransmitter metabolism, and energetics are used in pathological conditions to promote the survival of glioblastoma stem cells as well as HIV-1-CNS reservoirs.

## Establishment of the Viral Reservoirs: Link to Neuronal and Glial Metabolism

HIV-1 infection cannot be eradicated due to the early generation of viral reservoirs in several tissue compartments (Wong and Yukl, 2016). Despite common knowledge that the elimination of viral reservoirs is essential to cure HIV-1, the nature, characteristics, and mechanisms of latent and long-lasting HIV-1-reservoirs are unknown. Currently, the best-characterized viral reservoirs are different circulating subpopulations of CD4^+^ T lymphocytes; however, several groups propose that circulating viral reservoirs correspond to a poor representation of the real reservoir pool that is present in several tissues (Chomont et al., 2009; Soriano-Sarabia et al., 2014; Kandathil et al., 2016; Abreu et al., 2019).

In most cases, within the first few weeks of HIV-1 infection, the viral reservoirs are established in multiple tissues and anatomic compartments (Wong and Yukl, 2016). Compartmentalization of HIV-1 occurs as a result of differential selective pressures between tissues or restricted virus flow within organs (Borderia et al., 2007). Moreover, the different concentrations of antiretroviral drugs within compartments can result in divergent viral evolution (Zarate et al., 2007). It was shown that, in individuals diagnosed with HIV-1 infection, the pick level of HIV-1 replication was established in blood, oropharyngeal, and genital tract tissue, but not in the CSF (Pilcher et al., 2001). Furthermore, upon ART interruption, viral rebound was different between the blood and CSF, suggesting a differential compartmentalization (Garcia et al., 1999; Gianella et al., 2016; Mukerji et al., 2018). Early on in HIV-1-infection, the CNS becomes a unique compartment due to the presence of the BBB and the blood-CSF barrier, both of which restrict virus trafficking between the CNS and blood compartment, resulting in the rise of at least two separate HIV-1 compartments with different viral and cell evolutions (Smit et al., 2001; Wang et al., 2001; Ohagen et al., 2003; Abbate et al., 2005; Ritola et al., 2005; Yukl et al., 2013; Ganor et al., 2019). These differences in reactivation time and evolution can also be explained by the cell type infected, tissue microenvironment, access to ART, type of ART, pK distribution, proliferating cell properties, antigen dependency, or independent activation (Alexaki et al., 2008; Bingham et al., 2011; Pinkevych et al., 2015, 2016; Gianella et al., 2016).

Viral reservoirs are defined as cells with an integrated HIV-1 genome that have the capacity to silence viral replication, survive for extended periods of time, and become competent upon reactivation (Blankson et al., 2002). The best-described viral reservoirs are resting CD4^+^ T lymphocytes, including central memory T lymphocytes, transitional T lymphocytes, and effector memory T lymphocytes, which maintain the infection by homeostatic proliferation in central memory and transitional memory T lymphocytes (Lee and Lichterfeld, 2016). It was shown that activated T lymphocytes have a higher copy number of HIV-1-integrated DNA as compared to lymphocytes in the resting state of individuals with or without ART. Further characterization of the types of T lymphocytes indicate that HIV-1-DNA is present mostly in resting central memory and transitional memory T lymphocytes. These results indicate that both T lymphocyte populations are the circulating viral reservoirs (Chomont et al., 2009; Josefsson et al., 2013). Using the quantitative viral outgrowth assay (QVOA), it has been demonstrated that replication-competent viruses reside in transitional memory T lymphocytes even in samples obtained from HIV-1-infected individuals on long-lasting ART (Siliciano et al., 2003; Soriano-Sarabia et al., 2014). During early infection, CD4^+^ T lymphocytes are the major target cells (Haase, 1999). Next, within the first few weeks of primary HIV-1 infection, the virus population doubles every 6 to 10 h, resulting in infection of another 20 cells for each cell infected early on (Nowak et al., 1997; Little et al., 1999). During the symptomatic primary infection, the level of circulating virus in the blood (often >10^6^ to 10^8^/ml viral particles) and infected cell are high, and this initiates the compartmentalization early on in several tissues including the gut-associated lymphoid tissue (GALT). In 1995 studies by Wei et al. (1995) and Ho et al. (1995) showed viral evolution was a dynamic process because replacement of the wild-type virus can be achieved in 14 days, suggesting that viral replication and cell turnover can contribute to viral variability and the generation of reservoirs. In these studies, treatment with the HIV-1 reverse transcriptase and protease inhibitors (nevirapine and ritonavir) prevented infection of new cells but did not block virus replication in the cells already carrying integrated HIV-1 DNA. Both anti-viral drugs induced a decrease in plasma virus levels in the first 2 weeks of therapy. Further studies using mathematical models to calculate HIV-1 replication, half-life of replicating and reservoirs cells, as well as the potential contribution of tissue associated viral reservoirs indicates that ART is extremely effective in reducing systemic viral replication after 1–2 weeks after initiation by preventing new infections. However, a second phase of replication was detected that corresponded to the loss of long-lasting infected cells. However, a pool of tissue resident cells still maintained a slow decay in systemic replication. The authors estimate that 2.3 to 3.1 years are required to eliminate these HIV-1 cells from the tissue compartment, however, this cleaning does not mean eradication (Perelson et al., 1997). It was calculated that the resting T helper lymphocytes population in HIV-1 patients on ART may contain ~1 replication competent viral genome per 10^6^ CD4^+^ lymphocytes. Considering that the half-life on the latently infected memory T lymphocytes is around 4 years, 88 years of treatment would be required to reach a level of cure using current ART (Finzi et al., 1999; Shan and Siliciano, 2013; Hill, 2018).

HIV-1 enters the CNS early after primary infection (7–10 days), which contributes to the establishment of viral reservoirs in the CNS (Fischer-Smith et al., 2001; Valcour et al., 2012). CD14^+^CD16^+^ monocytes enter the CNS using physiological gradients of CCL2 from the brain and an upregulated expression of CCR2 on HIV-1-infected cells despite ART (Eugenin et al., 2006). More specifically, the mature CD14^+^CD16^+^ monocytes are strongly associated with CNS inflammation and HAND development (Williams et al., 2012, 2013). It was shown that in HIV^+^ CD14^+^CD16^+^ monocytes preferentially transmigrate across the BBB, and also junctional proteins JAM-A and ALCAM support their transmigration. Blocking of JAM-A and ALCAM by specific antibodies and the dual inhibitor of chemokine receptors CCR2/CCR5 prevent preferential transmigration of HIV^+^ CD14^+^CD16^+^ monocytes. Moreover, the surface of CCR2 is increased on HIV-1-infected CD14^+^CD16^+^ monocytes from individuals who manifested HANDs, independently of ART status, viral load, and CD4+ T lymphocytes count (Williams et al., 2014). The increase of CCR2 on CD14^+^CD16^+^ monocytes correlate with a higher level of HIV-1 DNA and neuronal damage, suggesting that CD14^+^CD16^+^ monocytes could have a key role in HAND pathogenesis and neuronal damage observed in infected individuals. After entering into the brain, HIV-1-infected monocytes differentiate into macrophages resulting in the release of hosts and viral components inducing infection of host cells such as microglia and astrocytes (Churchill et al., 2006).

Pericytes have a crucial role in the formation and maintenance of BBB integrity. Reduction in pericyte coverage is a typical feature of BBB impairment, observed in Alzheimer's disease (AD), multiple sclerosis (MS), amyotrophic lateral sclerosis (ALS), and also HAND (Sengillo et al., 2013; Winkler et al., 2013; Persidsky et al., 2016). The main cause of loss of pericyte integrity observed in these diseases is associated with loss of adhesion into the basal membrane and dedifferentiation of smooth muscle cells resulting in their detachment from endothelial cells (Persidsky et al., 2016). HIV-1 Tat also induce changes in pericytes, suggesting that the loss of pericyte coverage and their detachment from the endothelium contribute to loss of BBB integrity in HAND (Niu et al., 2014). Within the CNS, microglia, and macrophages are the main cells supporting HIV-1 replication, however, astrocytes also are infected but HIV-1 replication is minimal to undetectable. Even though only ~5% of the astrocytes become infected, their HIV-1-infection, but not replication, contributes to loss of BBB integrity (Churchill et al., 2006; Eugenin et al., 2011a). In pericytes, after HIV-1-infection, the viral replication decreased. Viral infection was demonstrated by Alu-PCR and infection was subjected to reactivation (Persidsky et al., 2016).

ART is a key factor in viral reservoir formation within the CNS. It has been demonstrated that initiation of ART less than 6 months after primary infection is associated with lower levels of T-cells activation and smaller circulating reservoir size during long-term therapy, supporting the hypothesis that reservoirs are formed early during infection (Jain et al., 2013; Abrahams et al., 2019). Moreover, by using mathematical models, it has been demonstrated that ART interruption with subsequent drug “washout” induces virus rebound (Hill et al., 2014, 2016; Pinkevych et al., 2015, 2016). Despite the efforts in re-using the success of ART to cure the disease, it is clear at this point that HIV-1-infected cells, including HIV-1 reservoirs, also have an alternative mechanism of survival, not only depending on silent or active replication. Some of these viral mechanisms are anti-apoptotic, metabolic, proliferating, and tissue sequestrating, and there is a variability between people and infected cells. All these mechanisms contribute to perpetuating the virus.

## HIV-1 and Glutamate Neurotoxicity—the Role of Glutamate Receptor Activation and Glutamate Metabolism

### HIV-1 and Glutamate Receptors

There are several viral and host proteins that have been described as mediating the over-activation of the Glu/GABA neurotransmission system (Eugenin et al., 2007; Ru and Tang, 2017). Some of these viral proteins are HIV-1 Tat and gp120. Gp120 is a viral envelope protein with neurotoxic activity. HIV-1 isolates from the CNS are mainly macrophage (M)-tropic (Gonzalez-Perez et al., 2012), but also the T-cell-tropic form was detected. The gp120 M-tropic and T-tropic glycoproteins can induce synaptodendritic degeneration by activation of chemokine co-receptors CXCR4 and CCR5, respectively (Kaul et al., 2007), localized on neurons and non-neuronal cells (Westmoreland et al., 2002). Gp120 induces a massive calcium release and changes in the morphology of neuronal mitochondria (Avdoshina et al., 2016; Rozzi et al., 2017). Also, gp120 is a glycoprotein with the ability to activate the NMDA receptors and increase the synaptic damage by overactivation. Direct mechanisms of neuronal injury induced by gp120/HIV via NMDAR involved NR2A and NR2B subunits based on enhanced NR2A- and NR2B-mediated EPSCs (Zhou et al., 2017). In the HIV/gp120-tg administration of the drug to mice, nitromemantine (a blocker of NMDAR), was protective against gp120 neurotoxic properties causing neuronal damage and synaptic loss. In the same studies, researchers demonstrated the crucial role of the NR3A subunit of NMDAR because the genetic overexpression of the NR3A subunit enhanced the synaptic injury in both gp120-tg and WT mice (Nakanishi et al., 2016). NMDAR phosphorylation in the cytoplasmic tail of this receptor has emerged as an important mechanism regulating its function and trafficking (Chen and Roche, 2007). Phosphorylation of Ser896 and Ser897 are crucial for channel activity but also for the promotion of trafficking from the Golgi to the plasma membrane (Scott et al., 2003). The T-cell-tropic gp120 promotes the trafficking and surface clustering of NMDAR, and this process was associated with the phosphorylation of the NMDA subunit NR1 at C terminal Ser896 and Ser897 (Xu et al., 2011). On the other hand, the M-tropic gp120 transiently decreased the level of pSer896 and pSer897 of the NR1 subunit, having the opposite effect on the T-tropic gp120 (Ru and Tang, 2016). Treatment with NMDAR and AMPAR antagonists prevents the effect of M-tropic gp120 on NR1 down-regulation, suggesting that phosphorylation of NR1 is dependent on synaptic activity and NMDAR activation (Ru and Tang, 2016). Moreover, both gp120 tropic forms present different neuropathological profiles—M-tropic gp120 has a weaker toxic property than T-tropic. Also, M-tropic (CCR5-preferring) is present in the early phase of HIV-1 infection, while T-tropic (CXCR4-preferring) in the late stage in patients with developed HAND (Bachis et al., 2010).

Tat is a potent excitotoxin and it stimulates NMDARs by direct cysteine–cysteine interaction with the extracellular domain of the receptor (Prendergast et al., 2002; Li et al., 2008). Moreover, Tat promotes the phosphorylation of the NMDA receptor and triggers the calcium efflux and receptor's stimulation (Haughey et al., 2001). Also, Tat can induce cell death in human neuroblastoma cells (SH-SY5Y) by stimulation of H_2_O_2_ release dependent on NMDAR activation (Capone et al., 2013). Amino acids 31–61 of Tat are necessary to cause neurotoxicity (Nath et al., 1996), and their cysteine residues are crucial for direct interaction with the NMDAR (Li et al., 2008). The role of Tat Δ31–61 was shown in studies with Tat Δ31–61 mutant protein where the modified protein was not able to bind to the NMDAR. Moreover, in the same studies, no interaction between Tat and NMDAR was observed (Li et al., 2008). The changes in the Tat protein structure prevented the interaction with the NMDAR, also the immune complex of the mutant Tat, nitrosylated Tat or anti-Tat antibody with NMDAR block neurotoxicity caused by NMDAR agonist (Rumbaugh et al., 2013). Tat as a transactivator of the HIV-1 long-terminal repeat (LTR) is necessary for viral replication (Moses et al., 1994; Taube et al., 1999). Tat causes neural injury by entering into the neurons by a low-density lipoprotein receptor-related protein-1 (LRP) (Liu et al., 2000; Eugenin et al., 2007). Tat binding to LRP on the neuron's membrane initiates the formation of a macro molecular complex among tat-LRP-PSD-95 (as an intracellular adaptor protein)-NMDAR, later nNOS is recruited to the complex with subsequent production of NO at the neuronal cell membrane (Eugenin et al., 2007). In this macro molecular complex, the NMDAR, especially the NR2A receptor subunit, play a crucial role in the process of Tat-induced apoptosis in human primary neurons (Eugenin et al., 2003; King et al., 2010). Application of the LRP antagonist, receptor-associated protein (RAP) completely blocked the Tat-dependent potentiation, which suggests that Tat potentiates NMDAR function via LRP (Krogh et al., 2014b). Application of Tat into human fetal neuron cell culture followed by calcium efflux mediated by excitatory amino acid receptors induced the calcium release via stimulation of IP_3_ receptor and activation of neuronal nitric oxide synthetase (nNOS). This process was abolished by inhibition of IP_3_ receptors, also had a neuroprotective effect from Tat toxicity (Haughey et al., 1999). Moreover, Tat potentiates the effect of NMDA on calcium release, by neuronal NMDAR sensitization so that the physiological level of Glu causes significant excitotoxicity and increases the intracellular calcium level. Our data indicates that blocking LRP, NMDAR, or nNOS activation reduces HIV-1 Tat internalization into neurons and prevents HIV-1 Tat-mediated apoptosis (Eugenin et al., 2007).

Also, HIV-1 protein gp120 acts synergistically with Tat and potentiates each protein's neurotoxic actions, promoting neuronal cell death (Nath et al., 2000). Most studies concerning the effect of Tat on NMDAR report that the changes in receptors function are acute (minutes to hours) (Haughey et al., 2001; Green and Thayer, 2016), while the neurotoxic properties of Tat occur after hours to days (Popescu, 2014). Tat-induced potentiation of the calcium release induced by NMDAR by 8 h then adapted, as later it was reported that it returned to the baseline by 24 h and dropped below control by 48 h (Krogh et al., 2014a).

Oligodendrocytes (OLs) are myelinating CNS cells. Their dysfunction leads to abnormal myelination, impairment of cell–cell signaling, and axon degeneration. OLs express iGluRs, including NMDA and AMPA receptors, and the expression of these receptors is highly heterogeneous (Salter and Fern, 2005). The NMDARs are mainly present on the myelin sheath, while AMPARs are equally distributed on the cell body (Micu et al., 2006). Also, the pattern of iGluRs distribution is different on immature and mature OLs; immature OLs represent a higher level of AMPARs and mature NMDARs, respectively. OL cells are also very sensitive to the toxic actions of Glu since the OL NMDARs are less susceptible to magnesium blockage; the level of Glu necessary to induce toxic effect in OLs may be lower than required for neurons (Karadottir et al., 2005). Activation of iGluR induced the accumulation of calcium in myelin cells in response to chemical ischemia. This effect was completely reversed by AMPA/KA antagonist (NBOX) in the oligodendroglial cell body but only moderately in myelin. In contrast, the broad spectrum of NMDAR antagonists (MK-801, 7-chloro-kynurenic acid, or d-AP5) decreased the level of calcium in myelin.

The massive release of Glu stimulated the NMDAR located on myelin and the accumulation of calcium inside myelin cells. The toxic action of Tat was also present in OL cells. In studies using transgenic mice expressing HIV-1 Tat, the primary corpus callosum and anterior commissure cell culture represent the increased level of OLs with aberrant morphology. Moreover, in the caudate-putamen, the myelin protein expression was abnormal. Tat protein caused the death of immature OLs and reduced myelin-like membrane production by mature OLs in a dose-dependent manner. Both toxic effects of Tat were reversed by the blocking of NMDARs by specific antagonist MK-801, while CNQX (AMPAR/KAR antagonist) only blocked the effect on immature OLs (Zou et al., 2015). This data confirms the results from Karadottir et al. (2005) that, depending on the stage of development (mature vs. immature), the pattern of iGluR is different.

In the current ART era, it still is a matter of debate whether viral proteins are produced within the brain. However, data from several laboratories including ours indicates that HIV-1 Tat, Nef, and gp120 are still produced and patients are developing antibodies to these proteins (Nath et al., 1987; Re et al., 1996; Rao, 2003; Eugenin et al., 2011b; Bachani et al., 2013; Nicoli et al., 2016). Besides, antiretroviral drugs do not affect the Tat protein level in HIV-1-infected patients, even when the viral blood load is low (Dickens et al., 2017). Thus, bystander damage by viral protein secretion is still a major concern mainly because ART blocks HIV-1 replication but is unable to reduce viral protein synthesis and secretion as well as associated inflammation and damage.

### HIV-1 and Glutamate Transporters

The major function of astrocytes is to support neurons by maintaining the balance of local ion concentration and pH homeostasis, regulating neurotransmitters level in extracellular space and clearing the metabolic waste (He and Sun, 2007). The dysfunction of the astrocyte network is a serious reason for the decline in neurons' survival. One of the main causes of neuron death is a massive release of Glu and NMDARs overactivation (Petralia, 2012; Wroge et al., 2012). Astrocytes prevent the neuronal loss by removing excess Glu from extracellular space by two Na^+^-dependent glutamate transporters (EAATs): GLAST/EAAT1 and GLT-1/EAAT2 (Rothstein et al., 1996). Loss of astrocytic Glu uptake and metabolism is one of the main factors responsible for neurotoxicity associated with neurodegenerative diseases, including HAND. It was shown that the most important factor in HIV-1-induced neurotoxicity is dysregulation of astrocytes Glu clearance (Potter et al., 2013). In monocyte-derived macrophages (MDM) HIV-1-infection up-regulate the EAAT-2 gene expression, but the transporter activity was decreased (Porcheray et al., 2006). Moreover, GS gene expression was decreased but the GS activity was increased (Porcheray et al., 2006). In HIV-1-positive people with HAND the astrocytic level of EAAT2 was reduced at the same time the extracellular level of Glu was increased (Xing et al., 2009; Borjabad et al., 2010). In the CNS a multiple factors cause the reduction of Glu transporters expression and in consequence astrocyte-dependent Glu clearance such as the pro-inflammatory mediators—tumor necrosis factor (TNF-α), interleukin (IL)-1β (Lindl et al., 2010) and HIV-1 viral protein—envelope glycoprotein (gp120), transactivator of transcription (Tat), and viral protein R (Vpr). The number of studies using cell cultures models has shown that gp120 significantly reduced the expression and function of Glu transporters, resulting in excessive accumulation of extracellular Glu in the synaptic cleft, overstimulation of Glu receptors and neurotoxicity (Nath et al., 2000; Wang et al., 2003; Visalli et al., 2007; Xu et al., 2011; Yang et al., 2013; Melendez et al., 2016; Zhou et al., 2017). HIV-1 protein, gp120 alters the neuronal function homeostasis and often cause synaptodendritic injury (Kaul et al., 2001; Visalli et al., 2007; Melendez et al., 2016; Zhou et al., 2017). In primary human astrocytes HIV-1 and gp120 by reducing the expression of EAAT2, impaired the clearance of Glu (Wang et al., 2003). In pharmacology studies of enzymes kinetics, the reduction in V_max_ of both glutamate systems (X_AG_ and X_c−_) was observed in striatal glial and neuronal cells from gp120 mice, with no effect on K_m_ for Glu (Melendez et al., 2016). These results suggest that gp120 reduces the density of Glu transporters in the mouse striatum without effect on Glu affinity to the transporters. Moreover, the total expression of GLT-1 protein was also altered in gp120 mice, which is involved in the development of HAND (Melendez et al., 2016). In Tat-transgenic mice the stimulus-evoked Glu release in the hippocampus and cortex was increased as compared to control animals, but GABA exocytosis was unaltered in the hippocampus and decreased in the cortex (Zucchini et al., 2013).

Protease inhibitors—Amprenavir (APV) and Lopinavir (LPV) caused the reduction of EAAT2 expression and diminished the intracellular concentration of Glu. LPV induced monocytes/microglia and astrocytes activation (Gupta et al., 2012), also in astrocytes LPV and APV increased Glu-dependent calcium efflux (Vivithanaporn et al., 2016). Moreover, the *in vivo* studies show a reduction in EAAT2 protein and mRNA level followed by the LPV treatment and suppression of the cortical level of L-glutamate and L-aspartate in conjunction with L-serine level reduction. The reduction of EAAT2 expression induced by LPV and APV on astrocytes reduces Glu extracellular clearance resulting in Glu overstimulation and calcium increase in glial cells (Vivithanaporn et al., 2016). Thus, a combination of HIV-1 infection and ART could also be additive negative effects in the glutamatergic system within the brain.

Tat specifically induced the macrophage/microglia activation and microglia-mediate neurotoxicity (Minghetti et al., 2004). Further it was shown that this process is caused by Tat-dependent activation of nicotinamide adenine dinucleotide phosphate (NADPH) oxidase (Turchan-Cholewo et al., 2009). NADPH oxidase is a key component of ROS production for oxidative burst and cell signaling pathways. The activation of NADPH oxidase in microglia induce the release of Glu (Barger et al., 2007). It was shown that, Tat induced the dose-dependent Glu release from microglial which is associated with increased expression of the X_c−_ glutamate-cystine antiporter, and this effect was blocked by NADPH oxidase and glutamate-cystine inhibitors (Gupta et al., 2010). Furthermore, the extracellular cystine is transported inside of the cell by the counter-transporter cystine and then transformed to GSH. In HIV-1-infection GSH levels are decreased enhancing further oxidative damage induced by HIV-1 (Wanchu et al., 2009; Ferrucci et al., 2012). GSH function is not only antioxidant but is also very important for a conjunction of various drugs and xenobiotics elimination (Rieder et al., 1995; Singh et al., 2017). Also the proper function of X_c−_ system can be interrupted by the upregulation of activity of nuclear factor erythroid 2-releated factor 2 (Nrf2), the main activator of the antioxidant response by neutralization of ROS accumulation and GSH depletion (Zhang et al., 2013). In astrocytes, the upregulation of xCT – X_c−_ system subunit altered by Nrf2 could be a potential source of excitotoxicity and neuron death induced by massive Glu release (Fogal et al., 2007; Bridges et al., 2012a,b). In astrocytes Tat protein induced the Nrf2 activation and X_c−_ system up-regulation (Mastrantonio et al., 2019). Moreover, it reduced the neuron viability in a mechanism dependent on Nrf2 activation and Glu release from astrocytes. This effect was blocked by X_c−_ system inhibitor—sulfasalazine, thus blockade of the transporter confirms the mechanism of neurotoxicity dependent on astrocytes activation (Mastrantonio et al., 2019). During oxidative stress conditions, cells can up-regulate the activity of Nrf2 to neutralize the ROS accumulation and not lead to GSH depletion (Niture et al., 2014). Astrocyte more resistant to oxidative stress in comparison to neurons which can strongly up-regulate the Nrf2 expression (Baxter and Hardingham, 2016). In response for cellular redox stare, Nrf2 migrates to the nucleus and binds to promoter region—antioxidant response element (ARE) of many antioxidant genes such as system X_c−_ subunit xCT, superoxide dismutase (SOD), glutathione peroxidase (GPX) (Niture et al., 2014). Treatment of human U373 astroglial cells with recombinant Tat increased the mRNA expression of several antioxidant enzymes such as X_c−_, Quinone Dehydrogenase 1 (NQO1), catalyze (CAT), Cu/Zn superoxide dismutase (SOD1 and SOD2), glutamate-cystine ligase (GCLC), and GPX (Mastrantonio et al., 2019). Also, U373 astroglial cells treated with Tat increased the nuclear Nrf2 level which was compatible with the transcriptional induction of antioxidant ARE genes, as well as decreased cell viability, suggesting that Tat-associated neurodegeneration could be caused by the X_c−_ system (Mastrantonio et al., 2019).

The peroxisome proliferator-activated receptors (PPARs) ligand-activated transcription factors belonging to the nuclear receptors for steroids, thyroid hormones, and retinoids. These receptors play major roles in cell differentiation, lipid homeostasis, and glucose regulation. Nowadays, the agonist of PPAR gamma (PPARγ) is promising treatment for neuroinflammation-related condition eg. Alzheimer's disease, Parkinson's disease, and stroke (Govindarajulu et al., 2018; Wen et al., 2018; Lee et al., 2019). It was demonstrated that PPAR attenuated the release of pro-inflammatory cytokines and oxidative stress promotors. Several laboratories demonstrated that PPAR agonist can be neuroprotective against HIV-1-associated neuroinflammation (Potula et al., 2008; Huang et al., 2014, 2015). In astrocytes and microglial PPARγ agonists, rosiglitazone and pioglitazone, prevented oxidative stress induced by cytokines and NO as well as GLT-1 expression induced by HIV-1 and gp120 exposure (Omeragic et al., 2017).

The astrocyte elevated gene-1 (AEG-1) is a novel protein involved in HAND development. AEG-1 is a multifunctional oncogene, identify as an HIV-1- and TNF-α-inducible transcript in astrocytes. Its higher expression has been demonstrated in HIV-1-infected astrocytes, as well as in astrocytes treated with gp120 and TNF-α (Kang et al., 2005). AEG-1 was studied in the cancer field, describe as oncogene implicated in the initiation of tumorigenesis, proliferation, metastasis, angiogenesis, and chemotherapy resistance of malignancies (Emdad et al., 2009, 2010; Lee et al., 2009; Chen et al., 2017). In malignant gliomas, expression of AEG-1 was high and associated with necrosis and neurodegeneration in correlation with reduced expression of EAAT2 (Lee et al., 2011). Moreover, the AEG-1 overexpression increased the expression of YY1, a transcriptional repressor of EAAT2 in astrocytes. The same effect was presented in the glioma cell line and is connected to glioma-associated necrosis (Lee et al., 2011; Vartak-Sharma et al., 2014). Present that inflammation-induced the expression of AEG-1 in astrocytes and loss of EAAT2 could contribute to neurodegeneration that has a neuroinflammatory manifestation, such as HAND. HIV-1-associated neuroinflammation is caused by cytokines and chemokines such as IL-1β, TNF-α which is followed by an imbalance in Glu homeostasis caused by EAAT2 and increased CCL2 production (Vartak-Sharma et al., 2014). Moreover, all the inflammation stimulus significantly increased AEG-1 expression in astrocytes and nuclear translocation induced by the nuclear factor (NF)-κB pathway which increases expression of NF- κB-responsive chemokine such as CCL2 (Vartak-Sharma et al., 2014).

### HIV-1 and Glutamate Metabolism Pathways

The main source of Glu overproduction in HIV-1 infection, includes the release of Glu from dying cells, disturbance in neurotransmitter clearance *via* impaired enzyme activity, and activated macrophages/microglia that also can release significant amounts of Glu into the extracellular space. Glu neurotoxicity is mainly caused by the up-regulation of Glu-generated enzyme—GLS isoform C in HIV-1-infected microglia. Also, the level of GLS isoform C was elevated in HIV-1-positive *post-mortem* brains which may be a compensatory effect induced by chronic exacerbate levels of Glu (Huang et al., 2011). The GLS C overexpression is also correlated with several neurocognitive and neurodegenerative disorders (Wang et al., 2017). The overproduction of Glu during HIV-1 infection in monocyte-derived macrophage (MDM) is caused by the release of GLS from the mitochondria followed by the generation of oxidative stress (Erdmann et al., 2009; Tian et al., 2012). In HIV-1-infected human MDM the Gln-dependent production of Glu was blocked by a GLS inhibitor (6-diazo-5-oxo-l-norleucine) and by the antiretroviral drug (zidovudine) (Zhao et al., 2004). Moreover, the excessive release of Glu from HIV-1-infected MDMs was inhibited by using *a* drugs that target N-acetylaspartylglutamate (NAAG) to specifically inhibit GLS (Erdmann et al., 2007). It was shown that Glu antagonist 6-diazo-5-oxo-L-norleucin (DON), a compound with GLS inhibition activity significantly decreased the Glu overproduction induced by HIV-1 and Glu-dependent neurotoxicity (Conti and Minelli, 1994; Zhao et al., 2004; Thomas et al., 2014). The Pro-drug of DON that is metabolized by carboxylesterases increasing the levels of the active drug mainly in the brain showing a higher CSF/plasma ratio (Nedelcovych et al., 2017). Furthermore, the new DON prodrug, JHU083, in a mouse model of HAND, reversed the deficits in spatial learning and working memory induced by HIV-1 infection. In biochemical tests JHU083 reduced extracellular level of Glu in CNS of mice infected with Eco-HIV, and decreased overstimulation of GLS in microglial cells (Nedelcovych et al., 2019).

HIV-1 viral protein R (Vpr) is known to promote virus replication in monocytes and differentiated macrophages (Connor et al., 1995). It was shown that soluble Vpr is detected in the CSF of HIV-1-positive patients, and it is a source of neurotoxicity and antioxidant system dysfunction (Patel et al., 2000; Ferrucci et al., 2012). Vpr transduced human derived macrophages and subsequent SILAC analysis indicates that around 50% of the proteins identified are involved in glycolytic and citrate pathways (Barrero et al., 2013). Next, labeling of glucose with Carbon-13 (^13^C) and subsequent treatment of macrophages transduced with Vpr indicates that labeled glucose was uptaked faster than untransduced cells. Measure of release of Gln, Glu, and α-KG in Vpr transduced cells indicate a faster glycolytic and TCA metabolism (Datta et al., 2016). Imbalance in glutaminolysis (deamination of Gln by GLS to Glu with subsequent conversion of Glu to α-KG) affects the production of antioxidants such as GSH and NADPH. Moreover, α-KG an intermediate product in Glu metabolism is an ROS homeostasis stabilizer. Thus maintenance of ROS homeostasis in HIV-1-infected macrophages is sufficient for cell survival (Datta et al., 2016). Vpr also up-regulates the GLS isoform C expression, resulting in an increased level of Glu in a media of HIV-1-infected macrophages (Erdmann et al., 2009; Datta et al., 2016).

### HIV-1 Metabolomics and Reservoir Survival

The analysis of circulating lipids of two HIV-1-infected populations, responding and non-responding to ART for 36 months, detected higher levels of HDL in the responding group and a high VLDL and low LDL in the non-responding group, suggesting that healthy lipids also are associated with better ART response (Palmer et al., 2014; Rodriguez-Gallego et al., 2018). Furthermore, examination of different groups of HIV-1-infected individuals separated by neurocognitive status (normal: stable neurocognitive impairments; worsening group: progression of neurocognitive impairments; and improving group: improving of their cognitive status) were analyzed by NMR. In the worsening group, the CSF levels of citrate and pyroglutamate were decreased, and the levels of glutamine and lactate were increased as compared to the normal cognition group. In contrast, stable and improved cognition showed decreased glutamine and glucose and increased citrate, pyroglutamate, and lactate as compared to the worsening group. This pattern suggests that the worsening group was associated with a higher glucose metabolism based on higher levels of creatine, suggesting higher energy demand; in contrast, the improving group was associated with a decrease in glycolysis and TCA cycle intermediates, suggesting a shift from aerobic to anaerobic metabolism as observed in the worsening group (Dickens et al., 2015). In HIV-1, it is known and well-described that the virus compromises most of the synaptic control of Glu as well as glial regulation of the glutamatergic system; however, the interpretation of these data was to increase synaptic compromise by overactivation of Glu receptors. Now, our data also indicate that high levels of extracellular Glu and Gln that are abound in HIV-1-conditons are a major source of energy to viral reservoirs (Castellano et al., 2019).

Overall, significant changes in brain volume have been observed, which may be related to synaptic compromise. In the pre-ART era, people with advanced disease had a significant reduction of cortical volume (Heindel et al., 1994; Peavy et al., 1994; Aylward et al., 1995) as compared to age-matched seronegative controls (Tate et al., 2010). The introduction of ART stopped the progression of brain volume loss in the thalamus, caudate, and cerebellum and caused cortical thinning in the frontal and temporal lobes and cingulate cortex (Sanford et al., 2018). Using MRS, the most common metabolic changes observed in HIV-1-positive patients is a decreased level of creatine (Cr); an increased level of choline (Cho) and myo-inositole (MI), which reflect neuroinflammation and microglial proliferation; and a decreased level of N-acetyl aspartate (NAA) and Glu as a markers of neuronal dysfunction and injury (Young et al., 2014). In patients before ART, the cortical level of inflammatory biomarkers Cho/Cr and MI/Cr was increased. In the same patients, the level of Glu/Cr in the basal ganglia was elevated and significantly declined after therapy initiation, suggesting a massive release of Glu from HIV-1-infected macrophages and microglia, as activated astrocytes function dysregulation. Introduction of ART within 5 years after infection attenuated the increase of these inflammatory markers (Young et al., 2014). Furthermore, in HIV-1-individuals under ART who manifest HAND, a lower ratio of NAA/Cr in frontal white matter, post cingulate, and precuneus was observed. Also, a decrease in the Glu/Cr ratio correlated with worse performance on verbal recall, psychomotor speed, and reaction time (Mohamed et al., 2018). In agreement with the previous data, a decreased level of NAA and Glu-Gln was also observed in the cortical gray matter during early HIV-1 infection, suggesting that HIV-1 causes neuronal and astroglial dysfunction soon after infection (Lentz et al., 2009).

In other studies, it has been demonstrated that in CSF of HIV-1-positive individuals who manifest HAND, the level of Glu was elevated, which suggests astrocyte activation—impairment of BBB integrity and Glu clearance. Moreover, in CSF, of all the examined individuals had an increased level of ketone bodies (BHBA and 1,2 propenodiol), which suggests the dysregulation of lipid metabolism (Cassol et al., 2014). This result is in line with our and other studies showing that HIV-1-infected cells can rapidly adapt and use an alternative sources of energy. In several pathologies, including cancer, glucose deficit results in the use of alternative sources of energy such as ketone bodies or amino acids (Laffel, 1999; Castellano et al., 2017).

CD4^+^ T lymphocytes are characterized as a circulating viral reservoir, which maintains survival by increased glucose transporter-Glut-1 expression and elevated glycolysis (Loisel-Meyer et al., 2012). It was shown that Glut-1 expression on CD4^+^ T lymphocytes is increased in HIV-1-infected individuals, and expression of Glut-1 could be a biomarker of CD4+ T lymphocyte activation in infected individuals (Palmer et al., 2014). Also, increased pyruvate delivery may be supported via the Glu-Gln cycle to cope with the high metabolic activity of infected cells. Delivery of a higher amount of ATP can enhance the proliferation of infected cells and induce viral replication (Loftus and Finlay, 2016).

Next to the CD4^+^ T lymphocytes, tissue macrophages can also establish HIV-1 reservoirs. First, HIV-1 replication occurs intracellularly in specific compartments described as a virus-containing compartments (VCCs), in which newly created virus particles sustain their infectious potential for extended periods of time (Gaudin et al., 2013), and, second, HIV-1-infected macrophages can survive the apoptotic response of the host cells against viral infection (Swingler et al., 2007; Castellano et al., 2017). It was shown that HIV-1-infected macrophages increase GSH synthesis to limit excitotoxicity and neutralize generated oxygen free radicals (Rimaniol et al., 2001). Furthermore, in latently infected macrophages, Bim, a highly pro-apoptotic negative regulator of Bcl-2, was upregulated and prevented Bcl-2-dependent sequencing of pro-apoptotic proteins in mitochondria. Furthermore, HIV-1 infection prevents the release of apoptosis-inducing factor (AIF) or cytochrome C from the mitochondria into the cytoplasm and apoptosome formation. Thus, HIV-1 infection and latency prevent apoptosis by compromising the formation of the transition pore in the mitochondria and by preventing the formation of the apoptosome (Castellano et al., 2017).

Previously we demonstrated that latent HIV-1-infected macrophages used unusual pathways to block apoptosis of infected cells by a Bim-mediated mechanism (Castellano et al., 2017). We characterized three different stages of HIV-1 infection in macrophages: an early stage (1–3 days post-infection) with increased HIV-1 replication; a middle stage (7–14 days post-infection) characterized by higher viral replication and high cell death, which affects mainly uninfected macrophages; and a late state (14–21 days post-infection) with minimal viral replication and cell death similar to the middle stage, resulting in the survival of a small population of infected macrophages. The stage of infection was associated with changes in mitochondrial metabolism where the basal oxygen consumption rate (OCR) was reduced in the early stage in contrast to middle stage where OCR was reduced and correlated with increased cell death. In last stage, where most surviving cells were infected, no future changes of OCR were observed. Moreover, blocking of mitochondrial complex V, which is responsible for ATP production, induced different cell responses in the early and late stages, but not in the middle stage where the level of apoptosis was the highest; this suggests that during minimal apoptosis respiration, ATP is reduced by the virus. Next, we established the metabolic changes in latently infected macrophages, which in contrast to CD4^+^ T lymphocytes, did not rely on glucose in the HIV-1 condition but used alternative sources of energy coming from the TCA cycle and accumulated lipids. Observed changes in macrophages metabolism suggest that compromised TCA is an additional source of carbon to accumulate lipids, which can be used as an alternative source of energy. The role of the TCA cycle was established by treatment of human macrophages with succinate, an intermediate in TCA that plays a crucial role in ATP production in mitochondria. Succinate induced lipid accumulation in HIV-1-infected macrophages in contrast to uninfected cells. Furthermore, we characterized that under HIV-1 conditions, latently infected macrophages are using Glu/Gln to provide α-KG and succinate for TCA cycle, whereas in uninfected macrophages, ATP is produced from fatty acids and glucose. Moreover, in the HIV-1 condition, blockade delivery of substrate for energy production (fatty acids, Gln, and glucose) induce a shift for use of alternative sources of energy. HIV-1-infected macrophages, in contrast to uninfected macrophages, cannot use alternative sources of energy, opening a unique opportunity to kill this type of reservoir. Furthermore, we demonstrated that latently HIV-1-infected macrophages used Gln, Glu, and α-KG to survive because the blocking of GLS or an amino acid transporter required for amino acid importation into the mitochondria induced a specific killing of the surviving HIV-1-infected macrophages (Castellano et al., 2019). Moreover, proinflammatory molecules, such as IFNγ and LPS, stimulate macrophage activation inducing glucose uptake with concomitant suppression of fatty acid uptake and oxidation (Vats et al., 2006). It was shown that released ROS and treatment with LPS increased the Glut-1 expression (Freemerman et al., 2014).

In this review, we presented the current knowledge about the role of Glu in NeuroHIV pathogenesis. Excessive release of Glu with concomitant blockade of reuptake and metabolism induce excitotoxicity and constant inflammation leads to HAND development. In a successful ART era, HIV-1 cannot yet be cured, due to viral reservoirs that are established in peripheral and CNS compartments. However, recent clinical observations have hypothesized that an early initiation of ART is crucial to a progressive contraction of the latent HIV-1 reservoir (“shrink”). This could possibly be accomplished with simultaneous strategies that activate (“kick” or “shock”) the latent reservoir and increase the clearance of virus-infected cells (“kill”), known as a “kick-kill” or “shock-kill” strategy (Chun et al., 1999; Archin et al., 2012; Van Lint et al., 2013; Pace and Frater, 2019; Edara et al., 2020). Although ART suppresses viremia in HIV-1 infected individuals, infected cells used Glu to maintain their survival and latent virus reservoirs. Moreover, Glu as a toxic molecule can induce viral rebound in latent infected cells. Thus, suppressing the overproduction of Glu shortly after infection may prevent neuroinflammation and successfully reduce the size of viral reservoirs that relay a significant amount of Glu to survive.

## Author Contributions

All authors listed have made a substantial, direct and intellectual contribution to the work, and approved it for publication.

## Conflict of Interest

The authors declare that the research was conducted in the absence of any commercial or financial relationships that could be construed as a potential conflict of interest.
